# Prevalence and molecular characterization of extended-spectrum β-lactamase-producing *Klebsiella pneumoniae* in Riyadh, Saudi Arabia

**DOI:** 10.4103/0256-4947.55306

**Published:** 2009

**Authors:** Mohammad H.M. Al-Agamy, Atef M. Shibl, Abdelkader F. Tawfik

**Affiliations:** From the College of Pharmacy, Pharmaceutics and Microbiology Department, King Saud University, Riyadh, Saudi Arabia

## Abstract

**BACKGROUND AND OBJECTIVES:**

Reports on extended-spectrum β-lactamases (ESBL) production by Enterobacteriaceae, and especially in *Klebsiella pneumoniae*, are few in Saudi Arabia. Therefore, we determined the prevalence of ESBL in *K pneumoniae* from Riyadh and characterized the predominant β-lactamase gene in these isolates.

**METHODS:**

A total of 400 *K pneumoniae* samples were isolated from two hospitals in Riyadh during 2007 and screened for production of ESBL using ESBL-E-strips and combined disk methods. PCR assay was used to detect *bla*_TEM_, *bla*_SHV_, and *bla*_CTX-M_ genes.

**RESULTS:**

Phenotypic characterization identified a high ESBL rate of 55% of *K pneumoniae* isolates. ESBL-producing *K pneumoniae* were PCR positive for SHV, TEM and CTX-M β-lactamase genes with prevalences 97.3%, 84.1% and 34.1%, respectively. Within the CTX-M family, two groups of enzymes, CTX-M-1 and CTX-M-9-like genes were found with prevalences of 60% and 40%, respectively.

**CONCLUSIONS:**

This study confirms the high rate of ESBL in *K pneumoniae* clinical isolates in hospitals in Riyadh. This study demonstrates the worldwide spread of *bla*_CTX-M_ genes. This first report of the presence of the *bla*_CTX-M_ gene in clincial isolates in Saudi Arabia is evidence of the continuing worldwide spread of this gene.

Extended-spectrum β-lactamase (ESBL)-producing *Escherichia coli* and *Klebsiella pneumoniae* have spread rapidly worldwide and pose a serious threat in healthcare-associated infections.^1^ ESBLs have spread threateningly in many regions of the world and now comprise over 300 variants (*http://www.lahey.org/studies*). A typical characteristic of ESBLs is their ability to hydrolyze oxyimino-cephalosporins and aztreonam while being inhibited by β-lactamase inhibitors.^2^,^3^ ESBLs arise by point mutations in genes for common plasmid-mediated β-lactamases TEM-1/-2 and SHV-1.^2^ The first ESBL SHV-2, which is a derivative of SHV-1, was reported from *ozaenae* in 1983.^4^ Several TEM-1 derivatives, however, confer ESBL properties and are prevalent in North America.^3^ In recent years, non-TEM and non-SHV plasmid-mediated ESBLs have been reported, mainly the CTX-M enzymes. The CTX-M family, first described in the early 1990s, is the most dominant ESBL among Enterobacteriaceae and is recognized as a rapidly growing family of ESBLs.^5^,^6^ The CTX-M enzymes are the predominant type of ESBL found in many regions of the world, including Asia, South America, Europe and Africa.^7^–^11^ The CTX-M enzymes form a rapidly growing family of currently over 69 enzymes (*http://www.lahey.org/studies*) categorized in five different groups called CTX-M-1, CTX-M-2, CTX-M-8, CTX-M-9 and CTX-M-25.^7^

In Saudi Arabia, few studies have been undertaken to determine the prevalence of ESBL-producing *K pneumoniae*^12^–^14^ However, β-lactamases were not molecularly characterized in these studies. Therefore, this study was devoted to determining the prevalence of ESBL in *K pneumoniae* and the molecular characterization of ESBLs in *K pneumoniae* isolates from Riyadh.

## METHODS

Four hundred *K pneumoniae* non-duplicate isolates were collected from hospitalized patients at two different hospitals in Riyadh, Saudi Arabia, during 2007. Susceptibility testing was performed by the disc diffusion method according to Clinical and Laboratory Standards Institute recommendations.^15^ The minimum inhibitory concentration (MIC) was determined by an E-strip test (AB BIODISK, Solana, Sweden) as described by the manufacturer. A laboratory control strain, *Escherichia coli* ATCC 25922, was used in the sensitivity test and in the MIC determination. Phenotypic detection of ESBL was carried out by two different methods: 1) the combined disc method using discs containing cefotaxime and ceftazidime with and without clavulanate (Becton Dickinson, USA) with the ESBL phenotype defined as an increase of ≥5 mm in the zone around the disc containing clavulanate compared to the zone of corresponding discs without clavulanate; and 2) using the E-test ESBL-strip (AB BIODISK, Solana, Sweden) with a ceftazidime gradient at one end and a ceftazidime plus clavulanic acid gradient at the other end. These strips were applied according to the procedure described by the manufacturer. ESBL was detected if the ratio of the MIC of ceftazidime to the MIC of ceftazidime plus clavulanic acid was ≥8. PCR methods were used to detect *bla*_TEM_, *bla*_SHV_, and *bla*_CTX-M_ using the primers and methods previously described.^9^,^16^,^17^ A further group-specific CTX-M PCR was performed to differentiate between the CTX-M-1, -2, -8 and -9 groups of enzymes using primers and methods previously described.^18^–^20^ The primers used in this study are listed in Table 1. All PCRs were conducted under standard conditions using plasmid DNA as templates.

**Table 1:** The primers used in amplification of β-lactamases genes

Gene	Primers	Nucleotide sequence	Position
TEM	T1	5'-ATT CTT GAA GAC GAA AGG GCC TC-3'	F
	T3	5'-TTG GTC TGA CAG TTA CCA ATG C-3'	B

SHV	NI1	5'-GCC CGG GTT ATT CTT ATT TGT CGC-3'	F
	NI2	5'-TCT TTC CGA TGC CGC CAG TCA-3'	B

CTX-M	CTX-MA	5'-CGCTTTGCGATGTGCAG-'3	F
	CTX-MB	5'-ACCGCGATATCGTTGGT-3'	B

CTX-M-1	ALA2	5'-ATGGTTAAAAAATCACTGCG-3'	F
	P2D	5'-CAGCGCTTTTGCCGTGTAAG-3'	B

CTX-M-2	CTX-M2GF	5'-TTA ATG ACT CAG AGC ATT C-3'	F
	CTX-M2GR	5'- GAT ACC TCG CTC CAT TTA TTG-3'	B

CTX-M-8	CTXM8GF	5'- TGA ATA CTT CAG CCA CAC G-3'	F
	CTXM8GR	5'-TAG AAT TAA TAA CCG TCG GT-3'	B

CTX-M-9	C-1	5'-AACACGGATTGACCGTCTTG-3'	F
	C-2	5'-TTACAGCCCTTCGGCGAT-3'	B

F= forward, B=backward

## RESULTS

ESBL phenotype was detected in 220 (55%) of 400 isolates. The antibiotic resistance rate and MIC distribution of ESBL-producing *K pneumoniae* are listed in Table 2. The resistance rate to cefotaxime, ceftazidime and amoxicillin/clavulanate were 97% (n=215/220), 95% (n=210/220) and 86% (n=190/220), respectively. A fourth-generation cephalosporin, cefepime, showed moderate activity (47%), but 4.5% (n=10/220) were resistant to cefoxitin and all ESBL-producing isolates were susceptible to imipenem. The resistance to cefoxitin in these isolates may be due to alteration in ompK35 or ompK36 and may not be due to AmpC enzymes because the MIC of β-lactam/β-lactamse inhibitors are markedly reduced in our isolates, but AmpC was not. In addition, the co-existence of other enzymes such as OXA may reduce susceptibility to β-lactam/β-lactamse inhibitors. Among non-β-lactam antibiotics, ESBL-producing isolates showed high resistance to gentamicin and amikacin (88.9% [200/220] and 773.% [170/220], respectively). However, ESBL-producing isolates showed a lower resistance rate of 11% to ciproflaxin.

**Table 2:** Minimum inhibitory concentration (MIC) of 220 extended-spectrum β-lactamase-producing *K pneumoniae* isolates.

Antibiotics	Distribution of MIC (μg/mL)																					Resistant	
	≥256	192	128	96	64	48	32	24	16	12	8	6	4	3	2	1.5	1	0.75	0.5	0.38	≥0.25	n	%
Amoxicillin/clavulanate	**40**	**30**	**10**	**35**	**30**	**25**	30	15	0	5	5	0	0	0	0	0	0	0	0	0	0	190	86.4
Piperacillin/tazobactam	**5**	**0**	**0**	**0**	10	0	15	10	11	14	26	14	45	27	43	0	0	0	0	0	0	5	2.4
Cefotaxime	**123**	**7**	**7**	**13**	**10**	**20**	30	4	0	6	0	0	0	0	0	0	0	0	0	0	0	215	97.7
Ceftazidime	**175**	**14**	**0**	**6**	**10**	**5**	**10**	**0**	0	0	0	0	0	0	0	0	0	0	0	0	0	210	95.5
Aztreonam	**160**	**25**	**10**	**5**	**5**	**0**	**10**	5	0	0	0	0	0	0	0	0	0	0	0	0	0	215	97.7
Cefepime	**35**	**0**	**5**	**0**	**0**	**0**	**5**	10	5	5	40	15	20	35	15	30	0	0	0	0	0	105	47.8
Imipenem	**0**	**0**	**0**	**0**	**0**	**0**	**0**	**0**	**0**	0	0	5	25	5	40	15	25	35	40	30	0	0	0
Cefoxitin	**1**	**0**	**3**	**1**	**1**	**2**	**1**	5	11	0	72	30	23	50	8	7	6	0	0	0	0	10	4.5
Gentamicin	**95**	**10**	**20**	**5**	**5**	**5**	**0**	**10**	**50**	3	7	6	2	1	1	0	0	0	0	0	0	200	88.9
Amikacin	**65**	**10**	**16**	**37**	**42**	15	10	1	2	5	5	1	8	6	4	3	0	0	0	0	0	170	77.3
Ciprofloxacin	**0**	**0**	**2**	**3**	**7**	**0**	**6**	**0**	**0**	**1**	**2**	**2**	**2**	5	9	1	5	15	40	20	100	25	11.4

MIC values in bold are in resistance range.

The PCR assays revealed that the prevalence of SHV, TEM and CTX-M genes was 97.3% (n=214/220), 84.1% (n=185/220), and 34.1% (n=75/220), respectively, in ESBL-producing isolates. Further PCR experiments to characterize CTX-M groups indicated that 45 (60%) of 75 CTX-M-producing isolates carry *bla*_CTX-M-1_ -like genes and 30 (40%) of 75 CTX-M-producers harbor *bla*_CTX-M-9_ -like genes.

## DISCUSSION

*K pneumoniae* is the most frequent ESBL-producing species worldwide. Production of ESBLs was detected in 220 (55%) of 400 clinical isolates of *K pneumoniae* isolated from hospital-acquired infections in Riyadh. There was no difference between the combined disk method and the E-test strip method in the phenotypic detection of ESBL for *K pneumoniae* isolates from Riyadh. Reports on ESBL production by Enterobacteriaceae, and especially in *K pneumoniae*, are few in Saudi Arabia. The overall prevalence of ESBL producers was found to vary greatly in different geographical areas and in different institutes in Saudi Arabia. In our study, the rate of ESBLs-producing isolates collected from two hospitals in Riyadh was relatively high (55%) and was similar to other rates reported from previous studies in Riyadh.^12^ However, lower rates for the prevalence of ESBL-producing *K pneumoniae* were detected in Abha and Al-Khobar (27.5% and 12.2%, respectively).^13^,^21^ A higher rate of ESBL production was observed in Riyadh (40% to 55%) followed by Abha (27.5%) and a lower rate was reported in Al-Khobar (12.2%). The variation is difficult to explain, but may be due to differences in the type and volume of consumption of antibiotics and differences in the time of collection of isolates. In Europe, a survey of 1610 *Escherichia coli* and 785 *K pneumoniae* isolates from 31 centers in 10 European countries found that the prevalence of ESBL in these organisms ranged from as low as 1.5% in Germany to as high as 39% to 47% in Russia, Poland, and Turkey.^22^ Higher figures of 30% to 60% have been reported for the South American countries of Brazil, Venezuela and Colombia.^23^–^25^ In Asia, ESBL production in Klebsiella has also been reported to be as low as 5% in Japan and ESBL rates in India for *E coli, K pneumoniae and K oxytoca* were 79.0%, 69.4% and 100%, respectively. ESBL-positive *E coli* rates were also relatively high in China (55.0%) and Thailand (50.8%) and Australia with higher rates of 20% to 50% in other parts of the continent.^3^,^26^,^27^ In Pakistan, the prevalence of ESBL-producing *K pneumoniae* was very high (70%).^28^ In the Arabian Peninsula, 23.5% of ESBL-producing *K pneumoniae* were identified as having the ESBL phenotype in Kuwait.^29^ However, in the United Arab Emirates, 36% of *K pneumoniae* was found to harbor the ESBL phenotype.^30^ In Jordan, 70% of *K pneumoniae* isolates express ESBL phenotypes and this prevalence is alarming.^31^

Resistance to β-lactams, especially third-generation cephalosporins and non-β-lactams, among clinical isolates of gram-negative bacteria is increasing worldwide.^32^,^33^ The overall resistance rate of ESBL-producing *K pneumoniae* isolates studied was alarmingly high to most antibiotics tested including gentamicin, amikacin, cefepime, and trimethoprim/sulfamethoxazole. Imipenem and cefoxitin followed by ciprofloxacin were the antibiotics most active against ESBL-producers. *K pneumoniae* isolates harboring ESBLs are significantly more often resistant to flouroquinolones.^34^,^35^ In the present study only 11.3% of ESBL-producing *K pneumoniae* were resistant to ciprofloxacin. In Iran, resistance to ciprofloxacin was found among 32% of the ESBL-producing *K pneumoniae* strains.^36^

TEM- and SHV-ESBL are derived from parental TEM-1 and SHV-1 by point mutations. TEM-1 and SHV-1 are non-ESBL; however CTX-M enzymes are not derived from non ESBL and consequently all CTX-M enzymes are ESBL. In the present study, the dominant β-lactamase was SHV with a prevalence of 97.3% followed by TEM and CTX-M with prevalences of 84.1%, and 34.1%, respectively. In the present study the prevalence of CTX-M enzymes was 34%, but the prevalence of CTX-M enzymes in a study under preparation was 85%. This may be because the present study was done on the isolates were collected during 2007 while in the other study the isolates collected during 2008. In addition to an insertion sequence (*ISEcp1*), which enhances the mobilization of the CTX-M gene, was detected in 60% of CTX-M-producing *Klebsiella* (study under preparation), while the prevalence of *ISEcp1* was relatively low (10%) in isolates included in the present study (Data not included). However, 32 (74%) of the 43 ESBL-producing isolates from Kuwait carried *bla*_CTX-M_.^37^ In Iran, the prevalence of *bla*_SHV_ and *bla*_TEM_ among ESBL-producing *K pneumoniae* was 69.6% and 32.1%, respectively.^36^ The prevalence of CTX-M in the present study was lower than the prevalence in Kuwait. However, the prevalence of TEM and SHV was higher than reported in Iran. The higher prevalence of CTX-M in Kuwait is due to most of the CTX-M being detected in non-Kuwaiti immigrants, mainly from South Asia, where CTX-M is endemic.^37^ Dissemination of CTX-M ESBL enzymes is worldwide.^7^ CTX-M-1 and CTXM-9 groups were detected in our isolates and no CTX-M-2 and CTX-M-8 groups were detected. The occurrence of *bla*_CTX-M-1_ -like genes (60%) was higher than *bla*_CTX-M-9_ -like genes (40%) in CTX-producing *K pneumoniae* in the present study. In Arab countries, the first description of CTX-M-15 was in Egypt and then in the United Arab Emirates and in Kuwait.^9^,^37^,^38^ CTX-M-15 is the predominant ESBL in Egypt, United Arab Emirates and in Kuwait.^9^,^37^,^38^ The present study reporting the CTX-M-15-like gene (CTX-M-1 group) and the CTX-M-14/18-like gene (CTX-M-9 group) is the first report of CTX-M genes in Saudi Arabia.

The *bla*_SHV_ gene was only found alone in 6.8% (n=15/220) of ESBL-producing isolates with elevated MIC for both cefotaxime and ceftazidime (≥256 mg/L). The presence of SHV β-lactamase alone suggests that these SHV genes are responsible for resistance to extended-spectrum cephalosporins in 6.8% of ESBL-producing *K pneumoniae* isolates. However, the *bla*_SHV_ gene was found to be associated with the *bla*_TEM_ gene in 56.8% (n=125/220) with three different patterns of resistance. Seventy-five of 125 (44%) isolates showed a higher MIC for both (≥256 mg/L), while 35 (20%) of 125 isolates exhibited an increase in MIC with cefotaxime and a decrease in MIC with ceftazidime, and 15 of 125 (7.2%) isolates exhibited a decrease in MIC with cefotaxime and an increase in MIC with ceftazidime. From this result either SHV or TEM or both are the ESBLs in 125 of 220 isolates producing ESBL. On the other hand, SHV β-lactamase was present with the CTX-M enzyme in 9.1% (n=20/220) of ESBL-producers. However, the SHV β-lactamase gene was present with both TEM and CTX-M in 25% (55/220). Thirty-five isolates belonged to the CTX-M-1 group with a higher MIC for cefotaxime and ceftazidime, while 20 isolates belonged to the CTX-M-9 group with a decrease in MIC with cefotaxime and an increase in MIC with ceftazidime.

CTX-M enzymes (CTX-M-1 like genes) were co-present with TEM in 5 isolates (2.27%) with an increase in MICs with cefotaxime and ceftazidime (≥256 mg/L). However, the CTX-M family of ESBLs has been increasingly detected worldwide.^5^ In the present study, the occurrence of CTX-M-producing *K pneumoniae* was relatively high (34.1%). In India, higher percentages (72%) of ESBL-producers harbor *bla*_CTX-M_ genes.^39^ In this study, we did not determine which β-lactamase was responsible for resistance to extended-spectrum cephalosporins because these genes were not sequenced. The DNA sequence of these genes must be done to know the type of β-lactamase gene and the prevalence of ESBL genes in ESBL-producing isolates in Saudi Arabia. To our knowledge, there is no published report of the discovery of *bla*_CTX-M_ genes in Saudi Arabia.

In conclusion, this study confirms a high rate of ESBLs in *K pneumoniae* in Riyadh, Saudi Arabia, and further demonstrates the worldwide spread of genes coding for CTX-M enzymes in clinical isolates. Most ESBL producers were resistant to oxyimino-cephalosporins and other non-β-lactam agents at high levels.
